# Activity profiling of peptidases in *Angiostrongylus costaricensis* first-stage larvae and adult worms

**DOI:** 10.1371/journal.pntd.0006923

**Published:** 2018-10-31

**Authors:** Karina M. Rebello, James H. McKerrow, Ester M. Mota, Anthony J. O´Donoghue, Ana Gisele C. Neves-Ferreira

**Affiliations:** 1 Laboratory of Toxinology, Oswaldo Cruz Institute, Fiocruz, Rio de Janeiro, Brazil; 2 Center for Discovery and Innovation in Parasitic Diseases, University of California, San Diego, La Jolla, CA; 3 Skaggs School of Pharmacy and Pharmaceutical Sciences, University of California San Diego, La Jolla, California, United States of America; 4 Laboratory of Pathology, Oswaldo Cruz Institute, Fiocruz, Rio de Janeiro, Brazil; Queen's University Belfast, UNITED KINGDOM

## Abstract

**Background:**

*Angiostrongylus costaricensis* is a relatively uncharacterized nematode that causes abdominal angiostrongyliasis in Latin America, a human parasitic disease. Currently, no effective pharmacological treatment for angiostrongyliasis exists. Peptidases are known to be druggable targets for a variety of diseases and are essential for several biological processes in parasites. Therefore, this study aimed to systematically characterize the peptidase activity of *A*. *costaricensis* in different developmental stages of this parasitic nematode.

**Methodology/Principal findings:**

A library of diverse tetradecapeptides was incubated with cellular lysates from adult worms and from first-stage larvae (L1) and cleaved peptide products were identified by mass spectrometry. Lysates were also treated with class specific peptidase inhibitors to determine which enzyme class was responsible for the proteolytic activity. Peptidase activity from the four major mechanistic classes (aspartic, metallo, serine and cysteine) were detected in adult worm lysate, whereas aspartic, metallo and serine-peptidases were found in the larval lysates. In addition, the substrate specificity profile was found to vary at different pH values.

**Conclusions/Significance:**

The proteolytic activities in adult worm and L1 lysates were characterized using a highly diversified library of peptide substrates and the activity was validated using a selection of fluorescent substrates. Taken together, peptidase signatures for different developmental stages of this parasite has improved our understanding of the disease pathogenesis and may be useful as potential drug targets or vaccine candidates.

## Author summary

*A. costaricensis* is a poorly studied nematode that causes abdominal angiostrongyliasis, a human parasitic disease. Peptidases perform several functions in the life cycle of parasites, including nutrition, differentiation and host invasion. The present study characterized the repertoire of peptidases in *A*. *costaricensis* lysates using a combination of peptide degradation screening using mass spectrometry and validation of the activity using fluorescent substrates and class-specific peptidase inhibitors. The results improved our understanding of the role of these peptidases in parasite biology, shedding light on the underlying disease mechanisms.

## Introduction

*Angiostrongylus costaricensis* is a zoonotic parasitic nematode that causes human abdominal angiostrongyliasis, a severe gastrointestinal disease. This helminth was first described in patients from Costa Rica in 1971 [1]. Abdominal angiostrongyliasis (AA) is currently widespread in Latin America [2, 3] and cases have also been reported in Africa and Europe [2].

Humans are incidental hosts who become infected following the ingestion of raw mollusks or unwashed vegetables contaminated with the mucous of mollusks containing third-stage larvae (L3) of *A*. *costaricensis*. The life cycle requires a mollusk (i.e.,slugs and/or snails) as an intermediate host and a rodent definitive host (i.e genus *Rattus* and cotton rats). Adult worms live inside the mesenteric arteries of infected rodents, where eggs laid by females are carried by the blood stream to the intestinal wall. The eggs hatch as first-stage larvae (L1), which migrate into the intestinal lumen and are further eliminated with the faeces. L1 are ingested [4] and/or penetrate through the mollusk tegument [5, 6] and two molts occur inside the intermediate host before the development of the infective L3 stage. To complete its life cycle, L3 larvae must be ingested by rodents, where they follow one of two alternative migratory routes during their development into adult worms: a lymphatic/venous–arterial pathway or a venous portal pathway. Both migratory pathways direct the worms to their final destination, the ileocecal region [3, 7, 8].

To date, definitive diagnosis of the infection requires a biopsy to find worms, eggs and/or larvae in histological sections. In the absence of parasitic structures, a probable diagnosis of AA is supported by a histological triplet involving the presence of eosinophilic infiltration in the intestinal wall, granulomatous inflammation and eosinophilic vasculitis [9]. Proposed noninvasive serological diagnostic tests have lacked sensitivity and specificity [10, 11]. Stool examination is not useful in diagnosing *A*. *costaricensis* because the eggs are not shed into human faeces. Furthermore, there is no specific and/or effective pharmacological treatment for this disease. Previous studies have shown that anthelminthics [12–16], antithrombotic drugs [17] and anti-inflammatory agents [18] are not effective against the nematode parasite. Therefore, the investigation of potential new diagnosis and/or treatment targets for AA is urgently required.

Peptidases are proteolytic enzymes that have been suggested as drug targets for parasitic diseases [19–22]. In parasitic helminths, peptidases are involved in host-parasite interactions, parasite immune evasion, life cycle transition and pathogenesis [23–26]. Using gel-based approaches, we have previously confirmed the presence of peptidase activity in L1 and L3 extracts. The gelatinolytic enzymatic activity of L3 larvae could be ascribed to metallo-peptidases, whereas the characterization of the protease activity of L1 larvae was inconclusive. Haemoglobinolytic activity due to aspartyl protease activity was also detected in the crude extracts of adult worms and in larval stages (L1 and L3) [27].

The present work aimed to provide a more detailed understanding of the proteolytic activity of adult worms and L1 lysates of *Angiostrongylus costaricensis*. To discover the proteolytic activity in these biological samples, lysates were incubated with an equimolar mixture of synthetic tetradecapeptide substrates. Both cleaved and uncleaved peptides were identified using tandem mass spectrometry and substrate specificity profiles were subsequently generated. This method, termed multiplex substrate profiling by mass spectrometry (MSP-MS) [28], has previously been used to discover protease activity in the excretion/secretion products from *Schistosoma mansoni* [29] and in the gut of *Schmidtea mediterranea* [30]. Further characterization of peptidase activity was performed using a panel of fluorescent peptide substrates and class-specific peptidase inhibitors.

## Methods

### Chemicals

The following protease inhibitors and peptide substrates were used in this study: Pepstatin A (Research Products, Mt. Prospect, IL, USA), E-64 (Merck Calbiochem, La Jolla, CA, USA), AEBSF (Sigma Aldrich, St Louis, MO, USA), and 1,10- phenanthroline (Acros Organics, Moris Plains, New Jersey, USA). H-Gly-Phe-AMC (7-amino-4-methylcoumarin), H-Tyr-AMC, H-Gly-Arg-AMC, H-Arg-AMC, Z-Arg-Arg-AMC, Z-Val-Arg-AMC, Z-Ala-Val-Asn-AMC, Glutaryl-Gly-Arg-AMC, Z-Val-Ala-Asp-AMC, Z-Val-Ala-Asp-AMC, Z-Val-Ala-Asp-AMC, Z-Val-Ala-Asp-AMC, Suc-Leu-Tyr-AMC and Z-Arg-Leu-Arg-Gly-Gly-AMC were purchased from Bachem (Torrance, CA, USA). Z-Phe-Arg-AMC was purchased from R&D Systems (Minneapolis, MN, USA). H-Ala-AMC, Z-Ala-AMC and Boc-Ala-Gly-Pro-Arg-AMC were purchased from Enzymes Systems Products (Livermore, CA, USA). N-Benzoyl-Phe-Val-Arg-AMC and N-Succinyl-Ala-Pro-Ala-AMC were purchased from Sigma. Z-Arg-Arg-Leu-Arg-AMC and Suc-Pro-Ser-Pro-AMC were from System Peptide Company (Pudong New Area, Shanghai, China). Boc-Leu-Arg-Arg-AMC (4-methylcoumarinyl-7-amide), Z-Ala-Ala-Asn-AMC and Z-Phe-Ala-AMC were from Peptide Institute (Ibaraki-Shi, Osaka, Japan). Suc-Leu-Leu-Val-Tyr-AMC and Suc-Gly-Pro-Leu-Gly-Pro-AMC were supplied by Peninsula Labs (Belmont, CA, USA). The internally quenched fluorescent substrate peptide substrate Mca-Gly-Lys-Pro-Ile-Leu-Phe-Phe-Arg-Leu-Lys(DNP)-DArg-NH2 was purchased from CPC Scientific (Sunnyvale, CA, USA). Stock solutions of substrate or inhibitor were dissolved in DMSO or water and diluted in buffer as required. In all cases, the DMSO concentration in the assays was less than 1% (v/v).

#### Ethics statement

All experiments on animals were approved by the Animal Ethical Committee at Oswaldo Cruz Foundation (CEUA Fiocruz license LW-26/15) and were conducted in accordance with the International Guiding Principles for Biomedical Research involving animals, as issued by the Council for the International Organizations of Medical Sciences.

#### Parasite material

Adult worms and L1 larval stage of *A*. *costaricensis* nematodes were obtained from the life cycle of the parasites, which is maintained in the Laboratory of Pathology (Oswaldo Cruz Institute, Fiocruz) using the intermediate snail host *Biomphalaria glabrata* and the definitive rodent host *Sigmodon hispidus*. *A*. *costaricensis* adult worms were obtained by careful dissection of mesenteric arteries of infected cotton rats [8]. L1 larvae were collected from faeces of these infected rodents, as previously described [27].

#### Cell lysate preparation

Adult worms (45 males or 40 females per biological replicate) were separately grinded for 5 min in chilled 1.5 mL microcentrifuge tubes containing resin (Sample Grinding Kit, GE Healthcare) and 200 μL of 40 mM Tris base with 0.1% Triton X-100. Samples containing L1 larvae (~ 120,000 larvae per biological replicate) were re-suspended in 250 μL of 40 mM Tris-HCl pH 6.8 with 0.1% Triton X-100 in 1.5 mL microcentrifuge tubes containing abrasive resin. Protein extraction was performed by a combination of grinding for 5 min on ice, followed by 10 freeze-thaw cycles in liquid nitrogen and room temperature. Cell debris were removed by centrifugation at 16,000 x *g* for 10 min at 4°C and the protein content in the supernatant was measured by BCA Protein assay (Thermo Scientific, Schwerte, Germany), using bovine serum albumin as standard.

#### Multiplex peptide cleavage assay

Lysates from female worms and L1 larvae (66 μg/mL) were assayed with a mixture of 228 tetradecapeptides (500 nM each) in 100 mM NaCl, 2 mM DTT and either 20 mM citrate-phosphate pH 3.0, 20 mM citrate-phosphate pH 5.0 or 20 mM Tris-HCl pH 8.0. These assays were performed in the presence or absence of the following inhibitors: a) pH 8.0 (1 mM AEBSF or 1 mM 1,10 phenanthroline); b) pH 5.0 (10 μM E-64); and c) pH 3.0 (1 μM pepstatin A). Twenty percent of the reaction volume was removed after 5, 15, 60, 240, and 1200 min incubation and the reaction was stopped by the addition of 8 M GuHCl. Samples were desalted with C18 LTS tips (Rainin) and injected into a Q Exactive Mass Spectrometer (Thermo) equipped with an Ultimate 3000 HPLC. Peptides were separated by reversed phase chromatography on a C18 column (1.7 um bead size, 75 um x 20 cm, heated to 65°C) at a flow rate of 400 nl min^-1^, using a 55-minute linear gradient from 5% B to 30% B, with solvent A: 0.1% formic acid in water and solvent B: 0.1% formic acid in acetonitrile. Survey scans were recorded over a 150–2000 m/z range at 70,000 resolution. MS/MS was performed in data-dependent acquisition mode with HCD fragmentation. Peak lists were generated using MSConvert (proteowizard.org) and data searches were performed against the library of 228 peptides using Protein Prospector software. All raw spectrum (.RAW) are available at the massIVE resource (https://massive.ucsd.edu/ProteoSAFe/static/massive.jsp; massIVE accession: MSV0000000082836). Tolerances of 20 ppm and 0.8 Da were used for parent and fragment ions, respectively. The following variable modifications were selected with a maximum of 2 modifications per peptide: amino acid oxidation (proline, tryptophan, and tyrosine) and N-terminal pyroglutamate conversion from glutamine. Protein Prospector score thresholds were set to 15 with maximum expectation values of 0.01 and 0.05 for protein and peptide matches, respectively. Peptide cleavage products were imported into iceLogo software v.1.2 [31] to generate protease substrate specificity profiles. Octapeptides (P4-P4ʹ) corresponding to the peptide cleavage products were used as the positive dataset, and octapeptides corresponding to all possible cleavage sites in the 228-member library were used as the background dataset (S1 File).

#### Assays using internally quenched fluorescent substrates

All assays were performed at room temperature in 0.1 M citrate-phosphate buffer pH 3.0 containing 100 mM NaCl, 2 mM DTT, 0.01% Tween-20 and 20 μM of Mca-Gly-Lys-Pro-Ile-Leu-Phe-Phe-Arg-Leu-Lys(DNP)-DArg-NH2. Assays were performed using three technical replicates of male and female lysates (150 μg/mL) or L1 lysates (125 μg/mL) in round bottom 96-microwell plates in the Spectra Max M5 spectrofluorimeter (Molecular Devices), with excitation at 328 nm and emission at 393 nm. Initial velocities (relative fluorescent units/sec) were calculated using the Softmax Pro software (Molecular Devices) and were converted to pmol/sec. Inhibition studies were performed using the same concentration of protein and substrates as outlined above, but with the addition of 1 μM of pepstatin A, 10 μM of E-64 or 1% DMSO.

#### Assays using AMC substrates

Enzyme assays were carried out in white 384-microwell plates (Corning Inc., Corning, NY) at room temperature using an EnVision Multilabel Plate Reader (PerkinElmer). Protein lysates (adult worms 150 μg/mL and L1 larvae 125 μg/mL) were assayed with a panel of fluorogenic substrates containing a C-terminal 7-amino-4-methylcoumarin (listed in S2 and S3 Tables) in 100 mM NaCl, 0.01% Tween-20, 2 mM DTT and either 100 mM citrate-phosphate buffer pH 5.0 or 20 mM Tris-HCl pH 8.0. The substrates (20 μL) were diluted in the same buffers at a final concentration of 100 μM. All assays were performed using three biological replicates. For the inhibition assay, aliquots of the lysates were pre-incubated for 30 min at room temperature, with the following inhibitors: 10 μM of E-64, 1 mM of AEBSF or 5 mM 1,10-phenanthroline. Since the inhibitors were dissolved in 1% DMSO, the positive control (lysate without inhibitor) was always assayed in the presence of 1% DMSO. Hydrolysis of the substrates was monitored, with excitation at 355 nm and emission at 460 nm.

## Results

### Adults worms

A global and unbiased substrate screen was used to uncover the peptidase substrate specificity in adult *A*. *costaricensis* worms. For these initial studies, protein lysates from female worms were used due to their larger size and sufficiently abundant protein levels; however, both male and female worm extracts were used for downstream validation assays. *A*. *costaricensis* protein was added to a mixture of 228 physicochemically diverse tetradecapeptides and cleavage of any of the 2,964 available peptide bonds within these peptides was detected by LC-MS/MS.

When assayed at pH 3.0, cleavage of 77 peptide bonds was discovered after 5 minutes incubation and, by 1200 minutes, a total of 556 cleaved peptide bonds were identified (S1 File). These data show that acid-acting peptidases present in *A*. *costaricensis* adults are capable of cleaving almost 20% of the available bonds in this tetradecapeptide library and are therefore likely to be able to degrade many protein substrates into short peptides. Aspartic acid peptidases are highly active at pH 3.0 and these enzymes are inhibited by pepstatin A. We therefore incubated worm lysate with this inhibitor prior to the addition of the peptide library. Under these assay conditions, we found a lower number of cleaved peptides at each time interval, when compared to the untreated assay, indicating that one or more aspartic acid peptidases are present in this worm sample.

**Fig 1:**
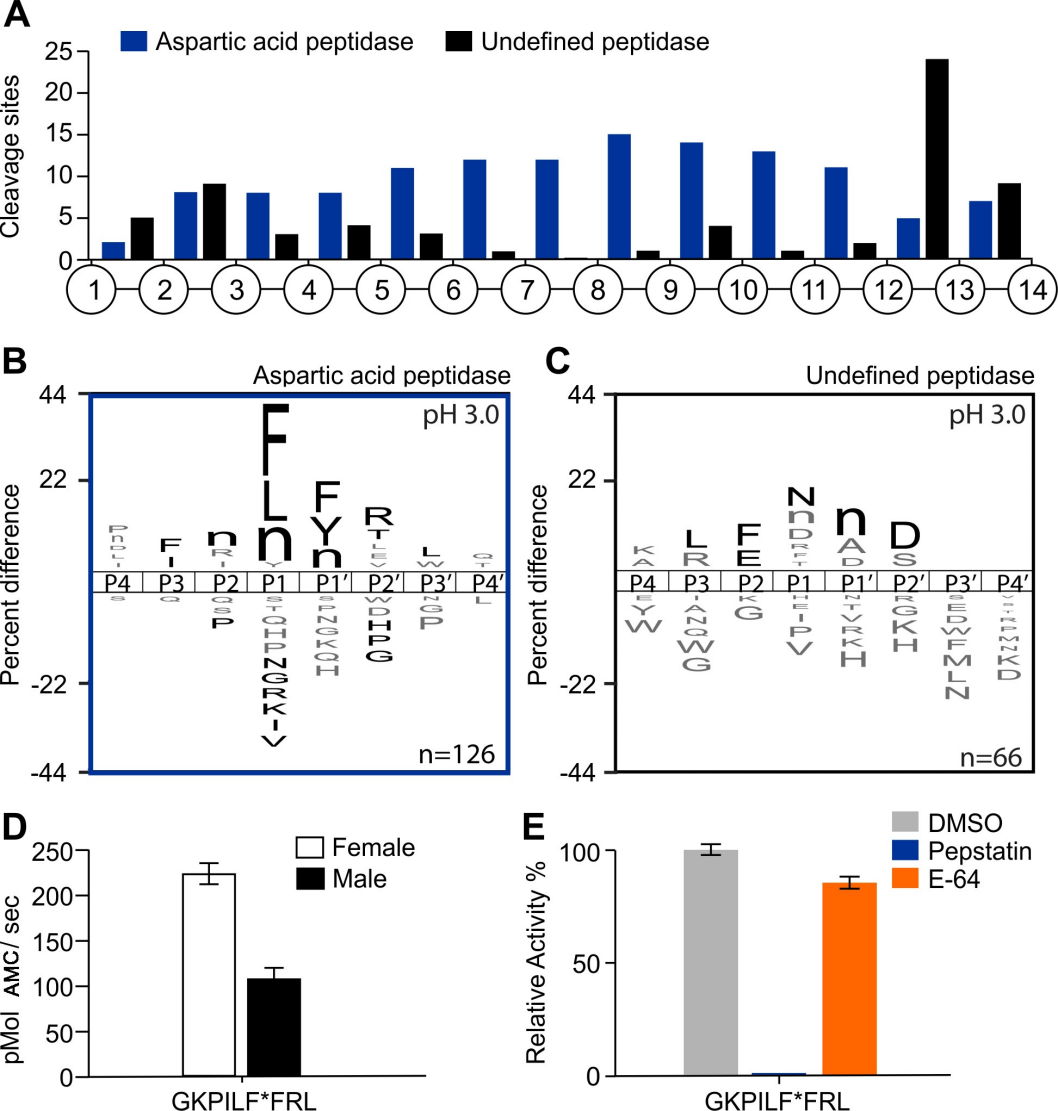
Characterization of the proteolytic activity of adult worm lysates at pH 3.0 using MSP-MS and an IQ-FRET substrate. A) Spatial distribution of the cleavage sites within the 14-mer peptide scaffold obtained with female lysates in the presence or absence of pepstatin (aspartic peptidase inhibitor). B-C) IceLogo representation showing the substrate specificity profiles obtained by MSP-MS following 15 minutes hydrolysis at pH 3.0 using female lysates (66 μg/mL). The amino acid ‘n’ corresponds to norleucine. Amino acids that are most frequently observed are shown above the axis, while less frequently observed ones are shown below. Residues highlighted in black are significantly (p ≤ 0.05) enriched relative to the frequency of these same amino acids in the peptide library. D) Adult worm lysates (female and male) (150 μg/mL) were incubated with internally quenched GKPILFFRL substrate (20 μM). Enzyme activity was expressed as picomoles of AMC per second. E) Female lysates were assayed with GKPILFFRL substrate in the presence of 10 μM E-64 and 1 μM pepstatin. The results are reported as mean ± standard deviation of three biological replicates.

A comparison of the cleaved peptide bonds that are generated in the presence and absence of pepstatin, allowed us to assign each cleavage site as being either the product of an aspartic acid peptidase or the product of other peptidases that are active at pH 3.0 but are not inhibited by pepstatin. Using only cleavage products that are detectable in the first 15 minutes of the assay of the DMSO treated assay, we discovered 126 products that are generated by aspartic acid peptidases as these are absent in the pepstatin A treated assay. In addition, 66 cleavage sites are present in both the DMSO and pepstatin A assays and therefore the products of one or more pepstatin-insensitive acid peptidases. The location of each cleavage site within the 14-mer peptides was determined. In general, the aspartic acid peptidases preferentially cleaved the 14-mer substrates between residues 5 and 12, indicating that they have greater endopeptidase activity than exopeptidase activity (Fig 1A). The pepstatin-insensitive peptidases generally cleaved between position 2 and 3, and between 12 and 14 indicating that these enzymes are likely to include at least one carboxypeptidase and one di-aminopeptidase.

A substrate specificity profile was generated for both types of enzyme activities. The aspartyl peptidase had a strong preference for hydrophobic amino acids Phe, Leu and norleucine (Nle) in the P1 site and no tolerance for Val, Ile, Lys, Arg, Gly or Asn. In the P1ʹ position, Phe, Tyr and Nle were preferred while Arg and Thr were frequently found at P2ʹ (Fig 1B). For the substrate specificity associated with the ‘undefined acid peptidase’ there was as preference for cleavage between Asn and Nle, with Asp commonly found at P2ʹ, Phe and Glu at P2 and Leu at P3 (Fig 1C).

The specificity of the *A*. *costaricensis* aspartic acid peptidase strongly correlates (Pearson correlation = 0.84) with the substrate specificity of human cathepsin D, an aspartyl peptidase that is also potently inhibited by pepstatin [32]. In fact, the standard fluorescent substrate for assaying cathepsin D consists of the following P4 to P3ʹ sequence, PILFFRL [33], which closely matches the optimal substrate sequence for the *A*. *costaricensis* aspartic acid. Therefore, to validate the substrate profiling we assayed the female lysate with the cathepsin D substrate and found it to be rapidly hydrolyzed (Fig 1D). In addition, we assayed a lysate of male *A*. *costaricensis* worms and found that this enzyme activity is also present, although the specific activity is decreased by 2-fold. Addition of pepstatin to the female lysate completely abolished this activity, while E-64, a broad-specificity cysteine peptidase inhibitor showed no statistically significant reduction in activity (2.83 ± 0.15%) (Fig 1E). Taken together, these studies show that one or more aspartic acid peptidases are active in the male and female worms at pH 3.0 and that this activity is inhibited by pepstatin. In addition, at least one enzyme with strong di-carboxypeptidase activity is also active at pH 3.0 in female *A*. *costaricensis* worms.

When the worm extracts were assayed at pH 5.0, cleavage of 84 peptide bonds was discovered after 15 minutes incubation and 512 peptide cleavage sites were detected by 1200 minutes (S1 File). Cysteine peptidases of the papain family are generally optimally active at pH 5.0 and these enzymes can be inhibited by E-64. Thus, we incubated the worm lysate with this inhibitor prior to the addition of the peptide library. Under these conditions, treated worm lysates exhibited a reduction in the number of cleaved peptides at all time intervals, when compared to the untreated control, indicating that one or more cysteine peptidases are present in this worm sample.

**Fig 2:**
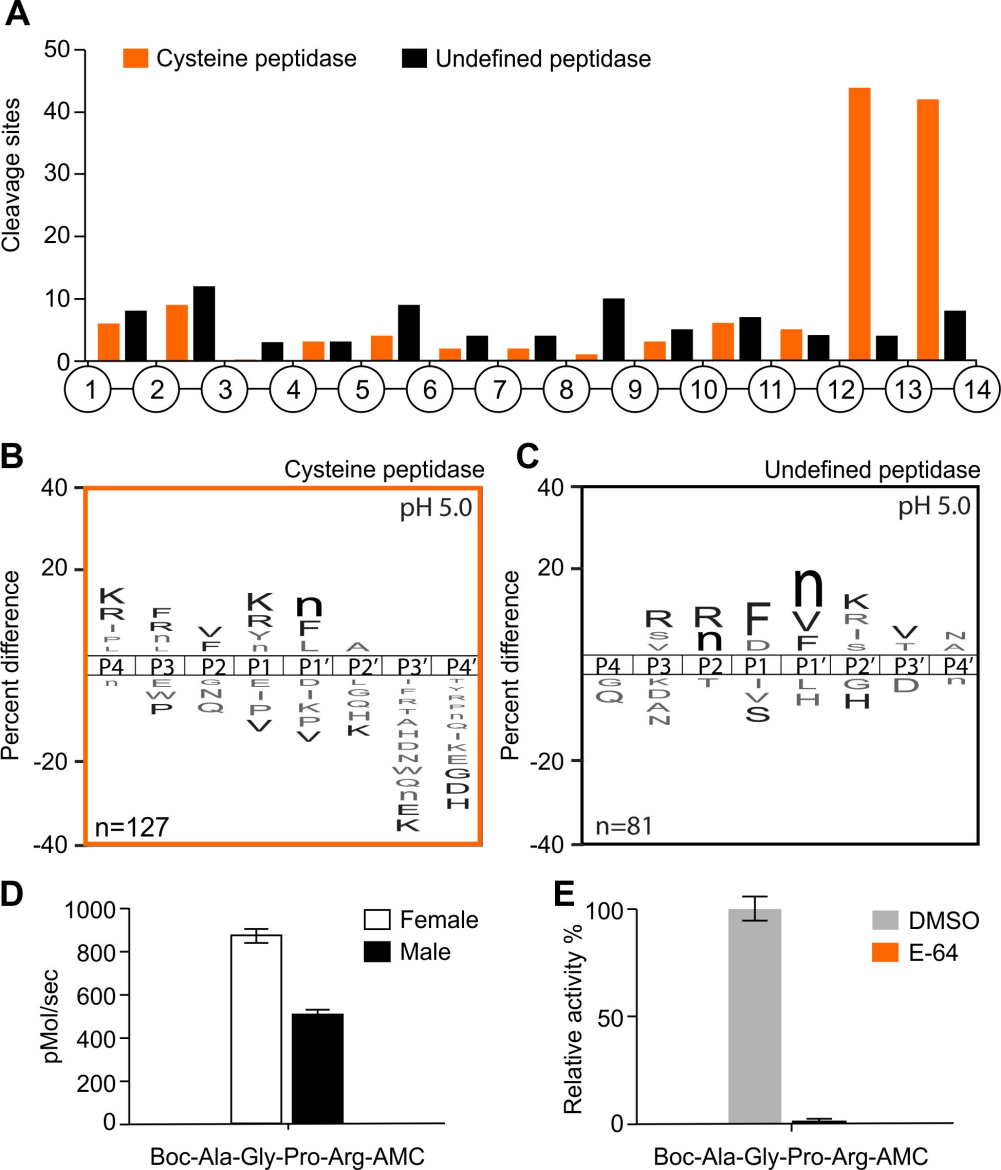
Characterization of the proteolytic activity of adult worm lysates at pH 5.0 using MSP-MS and AMC substrates. A) Spatial distribution of the cleavage sites within the 14-mer peptide scaffold obtained with female lysates in the presence or absence of E-64 (cysteine peptidase inhibitor). B-C) IceLogo representation showing the substrate specificity profiles obtained by MSP-MS following 60 minutes hydrolysis at pH 5.0 using female lysates (66 μg/mL). The amino acid ‘n’ corresponds to norleucine. Amino acids that are most frequently observed are shown above the axis, while less frequently observed ones are shown below. Residues highlighted in black are significantly (p ≤ 0.05) enriched relative to the frequency of these same amino acids in the peptide library. D) Adult worm lysates (female and male) (150 μg/mL) were incubated with Boc-Ala-Gly-Pro-Arg-AMC (100 μM). Enzyme activity was expressed as picomoles of AMC per second. E) Female lysates were assayed with the same fluorescent substrate in the presence of 10 μM E-64. The results are reported as mean ± standard deviation of three biological replicates.

Comparison of cleavage peptide events occurred in the presence and absence of E-64, allow us to determine each cleavage site as being either cysteine peptidase or other peptidases that are active at pH 5.0 but are not inhibited by E-64 (Fig 2A). Using only cleavage products identified after 60 minutes of the assay, 127 cleavage sites were detected as cysteine peptidases (Fig 2B) and 81 cleavage sites that are unchanged after E-64 treatment and are therefore the products of an undefined peptidase(s) (Fig 2C). Again, the location of each cleavage site within the 14-mer peptides was determined and the cysteine peptidases preferentially cleaved the 14-mer substrates between residues 12 and 14 while the other peptidases active at pH 5.0 hydrolyzed bonds within the 14-mer substrate without a clear preference for location (Fig 2A). These data indicate that the cysteine peptidases have mono- and di- carboxypeptidase specificity. A substrate specificity profile was generated for the cysteine peptidase that showed a strong preference for basic amino acids such as Lys and Arg in the P1 and P4 positions (Fig 2B). The substrate specificity associated with the ‘undefined peptidase’ was clearly different from the cysteine peptidase and showed a cleavage preference for norleucine (Nle) and Phe or Val in the P1´ position (Fig 2C).

The endopeptidase activity of adult worm lysates was also evaluated with 19 synthetic fluorogenic substrates at pH 5.0 (S1 Table). A substrate that was preferentially hydrolyzed by both female and male lysates contained the sequence Boc-Ala-Gly-Pro-Arg-AMC (Fig 2D). The location of Arg in the P1 position matches the substrate preference of the E-64 sensitive cysteine peptidases. When the female worm lysate was subsequently incubated with 10 μM of E-64 and assayed with Boc-Ala-Gly-Pro-Arg-AMC, the activity was completed inhibited (Fig 2E). These data show that *A*. *costaricensis* cysteine peptidases are active in both male and female worm lysates. These enzymes display broad substrate specificity but under this pH conditions, cleavage generally occurs near the carboxyl terminus of peptide and protein substrates thereby generating single amino acids and dipeptides.

We next assayed the female worm lysate at pH 8.0 to detect peptidases that are active at neutral pH and a total of 153 cleavage peptide bonds were identified (S1 File). Serine peptidases and metallopeptidases are generally active at neutral and basic pH, therefore, worm lysates were assayed in the presence and the absence of the serine peptidase inhibitor AEBSF and the metallopeptidase inhibitor 1,10-phenanthroline. Under each condition, we observed a decrease in the number of cleaved peptides at each time point, when compared to the untreated assay.

**Fig 3:**
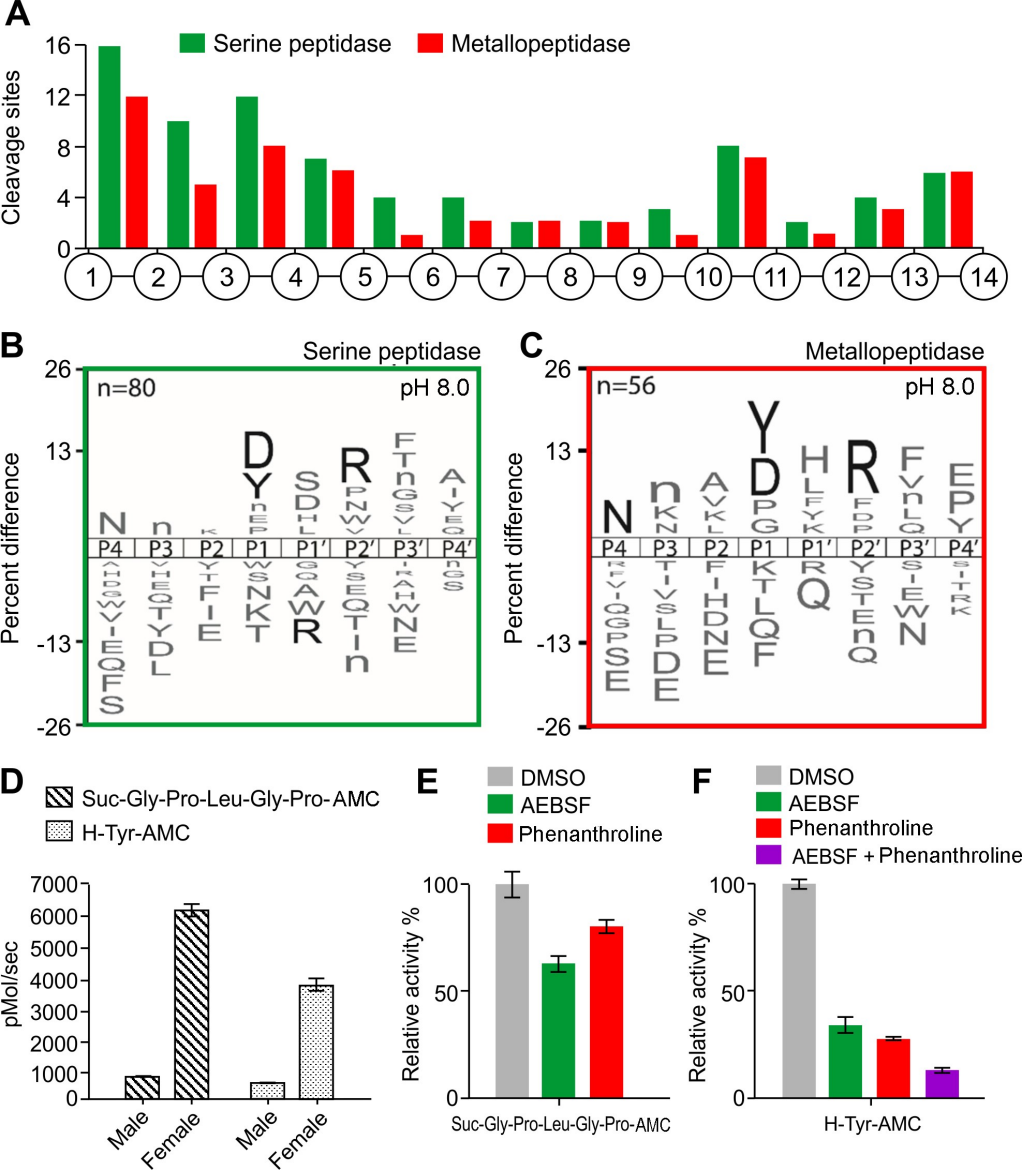
Characterization of the proteolytic activity of adult worm lysates at pH 8.0 using MSP-MS and AMC substrates. A) Spatial distribution of the cleavage sites within the 14-mer peptide scaffold obtained with female lysates in the presence of AEBSF (serine peptidase inhibitor) or 1,10 phenanthroline (metallopeptidase inhibitor). B-C) IceLogo representation showing the substrate specificity profiles obtained by MSP-MS following 240 minutes hydrolysis at pH 8.0 using female lysates (66 μg/mL). The amino acid ‘n’ corresponds to norleucine. Amino acids that are most frequently observed are shown above the axis, while less frequently observed ones are shown below. Residues highlighted in black are significantly (p ≤ 0.05) enriched relative to the frequency of these same amino acids in the peptide library. D) Adult worm lysates (female and male) (150 μg/mL) were incubated with Suc-Gly-Pro-Leu-Gly-Pro-AMC or H-Tyr-AMC (100 μM). Enzyme activity was expressed as picomoles of AMC per second. E) Female lysates were assayed with Suc-Gly-Pro-Leu-Gly-Pro-AMC in the presence of 1mM AEBSF or 5 mM 1,10 phenanthroline. F) Female lysates (150 μg/mL) were assayed upon H-Tyr-AMC in the presence of 1 mM AEBSF, 5 mM 1,10 phenanthroline or a mixture of these two inhibitors. The results are reported as mean ± standard deviation of three biological replicates.

The cleavage pattern within the 14-mer peptides after 240 min incubation at pH 8.0, in the presence of AEBSF or 1,10 phenanthroline is shown in Fig 3A. A total of 80 cleavage sites were associated with serine peptidase and 56 cleavage sites were shown to be generated by metallopeptidase. Serine peptidases showed strong preference for Asp and Tyr at P1, Arg at P2ʹ site and no tolerance for Arg at P1ʹ position (Fig 3B). Metallopeptidases also showed strong preference for Tyr and Asp at P1 site. In P4 position, Asn was preferred while Arg was frequently observed at P2ʹ site (Fig 3C).

The endopeptidase activity of both male and female worm lysates was also evaluated with 19 synthetic fluorogenic substrates at pH 8.0 (S1 Table). Several substrates and Suc-Gly-Pro-Leu-Gly-Pro-AMC most found to be hydrolyzed rapidly by peptidases in both samples. Using this substrate the specific activity of peptidases in female lysates was 7.3-fold higher than in males (Fig 3D). In female extracts, this proteolytic activity was reduced by 37 ± 2.89% in the presence of AEBSF and by 19 ± 2.08% with 1,10 phenanthroline (Fig 3E) indicating that both serine and metallo-peptidases can hydrolyze this substrate. The lysates were further assayed with mono- and di-peptide AMC substrates including Gly-Phe, Tyr, Ala, Arg or Gly-Arg (S2 Table), because many of the cleavage sites found in the tetradecapeptides substrate library were close to the amino termini indicating that one or more aminopeptidases were active in this sample (Fig 3A). Female lysates showed higher specific activity compared with male lysates, with a clear preference for a tyrosine residue in the P1 site. The aminopeptidase activity of female lysates was reduced by 65 ± 2.92% in the presence of AEBSF and by 72 ± 0.59% in the presence of 1,10 phenanthroline. When both inhibitors were included in the assay activity decreased by 86 ± 0.72% (Fig 3F). Taken together, these results indicate the presence of serine and metallo-peptidases in *A*. *costaricensis* adult worm lysates that have overlapping substrate specificity. 1,10 Phenanthroline is known to inhibit Zn-metallopeptidases by chelating the metal ions but it also has an affinity for Ca^2+^ ions. Therefore, the similarity in specificity between the serine and metallo-peptidases may to due 1,10 Phenanthroline inhibiting Ca^2+^ or Zn^2+^ dependent serine proteases in the *A*. *costaricensis* adult worm lysate.

### First larval stage (L1)

**Fig 4:**
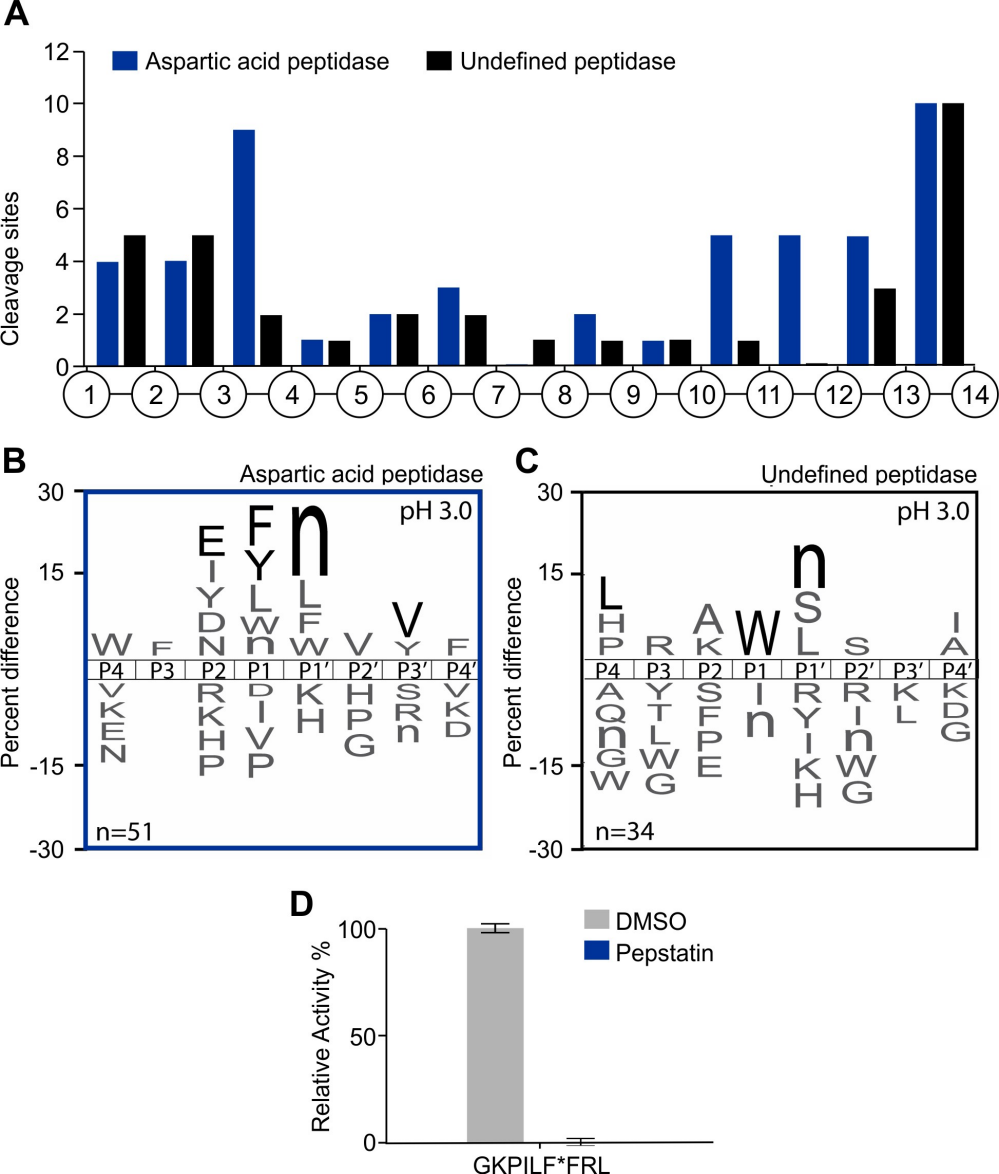
Characterization of the proteolytic activity of L1 lysates at pH 3.0 using MSP-MS and an IQ-FRET substrate. A) Spatial distribution of the cleavage sites within the 14-mer peptide scaffold obtained with L1 lysates in the presence or absence of pepstatin (aspartic peptidase inhibitor). B-C) IceLogo representation showing the substrate specificity profiles obtained by MSP-MS following 240 minutes hydrolysis at pH 3.0 using L1 larvae lysates (66 μg/mL). The amino acid ‘n’ corresponds to norleucine. Amino acids that are most frequently observed are shown above the axis, while less frequently observed ones are shown below. Residues highlighted in black are significantly (p ≤ 0.05) enriched relative to the frequency of these same amino acids in the peptide library. D) L1 lysates (125 μg/mL) were assayed with GKPILFFRL (20 μM) in the absence or presence of 1 μM pepstatin. The results are reported as mean ± standard deviation of three biological replicates.

We next evaluated peptidase activity in *A*. *costaricensis* L1 lysate to determine if these enzymes differ from the peptidases detect in adults. When L1 lysate was assayed at pH 3.0 with the tetradecapeptides library, cleavage of 11 peptide bonds was discovered after 5 minutes incubation and, by 1200 minutes, a total of 138 cleavage peptide bonds were identified (S1 File). When this same assay was performed in the presence of pepstatin, a decrease in the number of cleaved peptides occurred at each time interval, although not all peptide cleavage sites were sensitive to pepstatin. Using only cleavage products that were detectable after 240 minutes incubation, 51 were generated by aspartic acid peptidases (Fig 4B) and 34 are unchanged after pepstatin treatment, therefore representing the products of undefined peptidases (Fig 4C). Aspartic acid peptidase preferentially cleaved the 14-mer peptides between residues 3 and 4 and between residues 10 and 14 while the undefined peptidase frequently cleaved between position 1 and 3 and between 13 and 14 (Fig 4A). The location of the cleavage sites differs from what was seen with the aspartyl peptidases present in the adult lysate (Fig 1A). However, the overall substrate specificity preference was similar to the adult lysate peptidases with cleavage occurring between two hydrophobic residues such as Phe and Tyr at P1 and norleucine (Nle) at P1ʹ. In addition, these enzymes preferred Glu at P2 and Val at P3ʹ (Fig 4B). For the substrate specificity associated with the ‘undefined acid peptidase’ there was a preference for Trp at P1, Leu at P4 and Nle at P1ʹ (Fig 4C).

We predicted that the acid peptidase may also hydrolyze the internally quenched fluorescent substrates that was designed for human cathepsin D and could be readily hydrolyzed by aspartic acid peptidases in the *A*. *costaricensis* adult lysate. When this substrate was assayed with L1 lysate at pH 3.0, cleavage was observed, and this activity was completely inactivated by pepstatin (Fig 4D). These data show that many of the peptidases active in L1 are likely aspartic acid enzymes, as already observed for adult worms.

**Fig 5:**
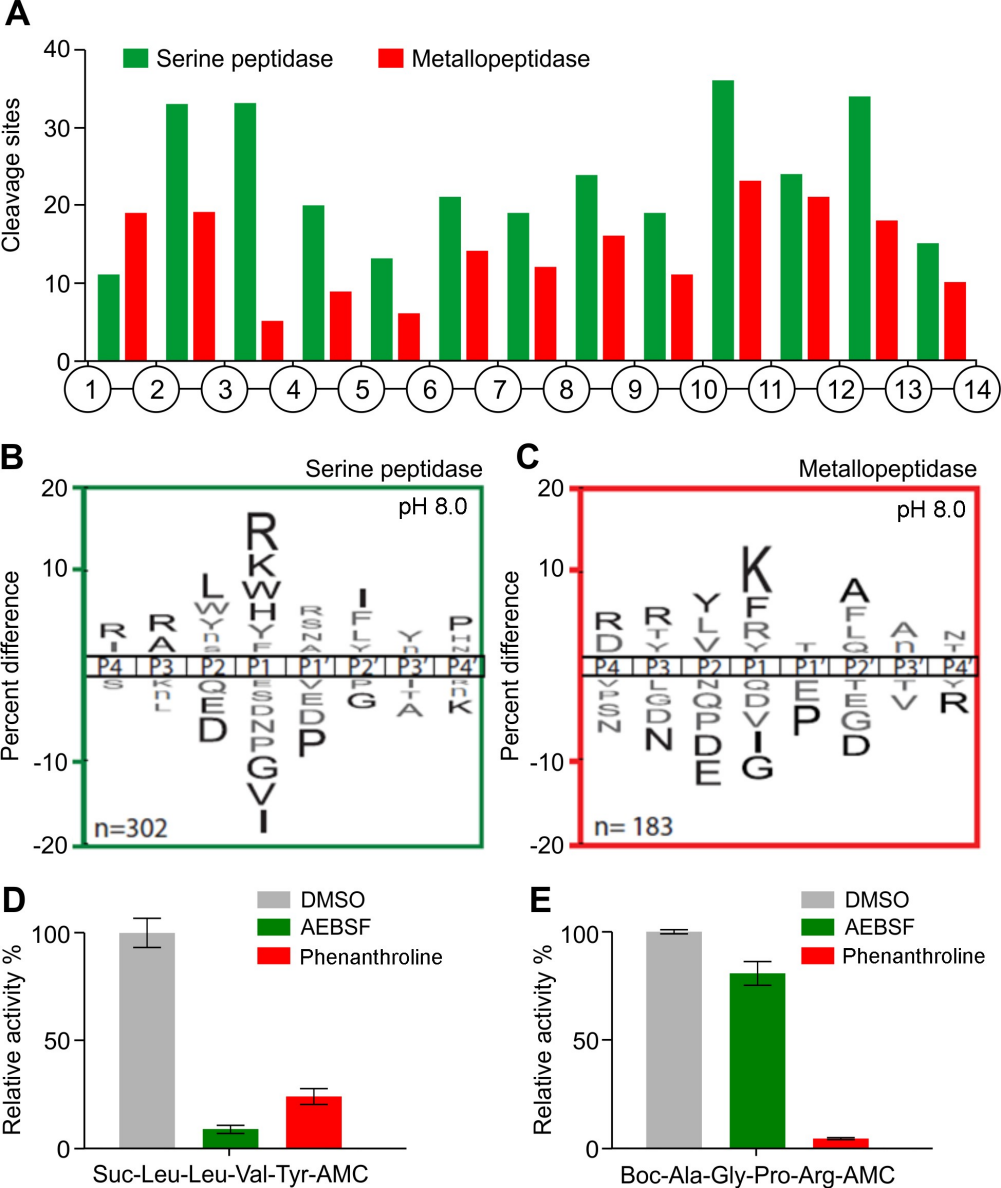
Characterization of the proteolytic activity of L1 lysates at pH 8.0 using MSP-MS and AMC substrates. A) Spatial distribution of the cleavage sites within the 14-mer peptide scaffold in the presence or absence of AEBSF (serine peptidase inhibitor) or 1,10 phenanthroline (metallopeptidase inhibitor). B-C) IceLogo representation showing the substrate specificity profiles obtained by MSP-MS following 15 minutes hydrolysis at pH 8.0 using L1 larvae lysates (66 μg/mL). The amino acid ‘n’ corresponds to norleucine. Amino acids that are most frequently observed are shown above the axis, while less frequently observed ones are shown below. Residues highlighted in black are significantly (p ≤ 0.05) enriched relative to the frequency of these same amino acids in the peptide library. D) L1 lysates (125 μg/mL) were incubated with Suc-Leu-Leu-Val-Tyr-AMC (100 μM) in the presence of 1 mM AEBSF or 5 mM 1,10 phenanthroline. E) L1 lysates (125 μg/mL) were assayed upon Boc-Ala-Gly-Pro-Arg-AMC (100 μM) in the presence of 1 mM AEBSF or 5 mM 1,10 phenanthroline. The results are reported as mean ± standard deviation of three biological replicates.

When the L1 lysate was assayed in pH 8.0, a total of 634 cleavage peptide sites were detected within the 14-mer peptides after only 15 minutes. With extended incubation for up to 1200 minutes, 1289 unique peptide bonds were hydrolyzed (S1 File). Similarly, to the pH 8.0 studies performed with the adult lysate, the L1 lysate was pre-treated with either AEBSF or 1,10 phenanthroline. A total of 302 sites were found to be the product of serine peptidases, while 183 peptide bonds were cleaved by metallopeptidases. In general, the distribution of cleavage sites was similar for both classes of enzymes, and the serine peptidase was dominant, with the notable exception of the bonds between amino acid 1 and 2, where metallopeptidase activity prevailed (Fig 5A). The serine peptidases showed strong preference for basic or bulky residues (Arg, Lys, His) and Trp at P1 position and no tolerance for Gly, Val and Ile. At the P2 site, Leu was preferred and no tolerance for Glu and Asp. At the P3 position, Arg and Ala were preferred, while Arg and Ile were frequently found at P4. At the P4ʹ site, Pro was preferred while this same amino acid was not tolerated at P1ʹ (Fig 5B). Metallopeptidases showed strong preference for Lys and Phe at the P1 site and Ala at P2ʹ (Fig 5C).

Additionally, the endopeptidase activity of L1 lysates was also evaluated with a selection of synthetic fluorogenic substrates at both pH 5.0 and 8.0 (S3 Table). Suc-Leu-Leu-Val-Tyr-AMC and Boc-Ala-Gly-Pro-Arg-AMC substrates were preferentially hydrolyzed at pH 8.0 and all substrates that where hydrolyzed at pH 8.0 were also hydrolyzed at pH 5.0. As the specific activity was always higher at pH 8.0, we chose to focus our efforts on assays performed at pH 8.0. The proteolytic activity observed with Suc-Leu-Leu-Val-Tyr-AMC was inhibited by 91 ± 1.72% in the presence of AEBSF and by 75.0 ± 3.66% when using 1,10-phenanthroline (Fig 5D). Cleavage of Boc-Ala-Gly-Pro-Arg-AMC was slightly reduced upon treatment with the AEBSF (9.2 ± 2.5%), and it was inhibited by 96.0 ± 0.25% in the presence of 1,10-phenanthroline (Fig 5E). These results indicate that both serine and metallo-peptidases are responsible for cleavage of Suc-Leu-Leu-Val-Tyr-AMC and while metallopeptidases cleave the Boc-Ala-Gly-Pro-Arg-AMC substrate.

Taken together, our data indicate that multiple proteases are present in L1 and adult worm lysates of *A*. *costaricensis*; they are capable of cleaving a diverse set of peptide bonds over a broad pH range.

## Discussion

The present work shows that *A*. *costaricensis* expresses many different peptidases in adult and larval stages. Proteolytic enzymes are receiving increasing attention as potential therapeutic targets or as diagnostic markers for various diseases [34–36]. In helminths, they have been implicated in a broad range of biological process [35, 37–39]. Very little is known about the function of proteolytic enzymes in *A*. *costaricensis*, therefore biochemical characterization of their proteolytic enzymes may provide insights into parasite-host interaction mechanisms involved in the establishment and development of abdominal angiostrongyliasis.

Using a wide variety of peptide substrates and several classes of specific peptidase inhibitors, aspartic acid, cysteine, serine and metallo-peptidases from *A*. *costaricensis* were detected and partially characterized. The cysteine peptidase specificity in adult worms matched papain-type peptidases found in mammalian cells. Human cathepsin B and L are inhibited by E-64 and show preference for positively charged amino acids at the P1 position, in addition to hydrophobic amino acids at the P2 position [40]. Cysteine peptidases have been implicated in multiple functions that are essential to the biology of parasitic organisms, such as antigen presentation, digestion, immune invasion, hemoglobin hydrolysis and host invasion [41]. Cathepsins B and L perform the majority of digestive function in helminths [37, 41] and RT-PCR data analysis of *Angiostrongylus cantonensis* showed that cysteine peptidase transcripts are present in larval stages and adult worms [42]. In addition, similarly to cathepsin B, which was shown to have carboxypeptidase activity [43], most activity attributed to cysteine peptidases in *A*. *costaricensis* worm extract occurred at the C-terminal side of the substrates. Human cathepsin B and L do not tolerate Pro at the P2 position and therefore it is unexpected that the fluorescent substrate Boc-Ala-Gly-Pro-Arg-AMC was rapidly cleaved by the worm cysteine peptidases. However, it is possible that *A*. *costaricensis* also express cysteine peptidases with a substrate specificity similar to human cathepsin K, as this enzyme preferentially cleaves substrates that contain proline at P2 and Arg at P1 [43]. A gut associated cathepsin B cysteine peptidase was identified in juvenile and adult worms of *Angiostrongylus cantonensis*, suggesting that these enzymes are involved in nutrition [44, 45]. The biological function of *A*. *costaricensis* cysteine peptidases remains unknown, but this study has confirmed that these enzymes are active in the adult worm.

Aspartic peptidases are a group of evolutionarily conserved proteolytic enzymes that have been characterized in many parasites [35, 46, 47]. Aspartic peptidases trigger a multienzyme cooperative cascade of hemoglobin proteolysis inside the guts of nematodes and other parasites. They are responsible for cleaving the intact hemoglobin before it can be further processed by additional digestive peptidases [35]. Using a fluorescence substrate developed for human cathepsin D, this study confirmed that *A*. *costaricensis* adult worms and L1 larvae express one or more enzymes that are functionally related to human cathepsin-D. These enzymes are inhibited by pepstatin and preferentially hydrolyze protein between two hydrophobic amino acids. Our previous data showed that aspartic peptidase inhibitors could block hemoglobin degradation by *A*. *costaricensis* [27], therefore this proteolytic activity is likely to be required for parasite nutrition by the nematode. Interestingly, initial activity profiling of ticks *Ixodes ricinus* gut lysates indicated that hemoglobinolysis is optimal at acid pH, suggesting that proteolysis is mediated by peptidases belonging to the aspartic and/or cysteine peptidase classes which are known to operate optimally at acid pH [48]. Blood-feeding pathogens digest hemoglobin as a source of nutrition but little is known about this process in *Angiostrongylus costaricensis*. In helminths, hemoglobin usually is digested by a cascade of aspartic and cysteine peptidases [49–51]. In adult schistosome worms, for example, digestion of blood requires a combination of cysteine and aspartic peptidases [35, 52].

In female worms, there are at least two serine peptidases which are active at pH 8.0. The specificity profile of the AEBSF-sensitive enzymes showed a preference for Asp or Tyr at P1. It is unlikely that the same enzyme is hydrolyzing both amino acid residues. The serine peptidase cleaving substrates showing Asp at the P1 site is likely to be an endopeptidase; the other enzyme is an aminopeptidase that prefers amino terminal Tyr residues. Interestingly, the specificity profile of serine peptidase activity in worms differs from serine peptidases identified in L1 larvae, which were described as trypsin-like enzyme(s). The activity profiles of metallopeptidases in female and L1 lysates were also different. Metalloaminopeptidases and serine trypsin-like were characterized in excretory/secretory products of *Anisakis simplex* larvae using fluorogenic substrates. These enzymes were postulated to be involved in host tissue penetration [53]. Similarly, a metalloaminopeptidase from *Brugia pahangi* was found to participated in the moulting process [54]. Serine and metallo-peptidases have been identified in the secretome from the fourth-stage (L4) larvae and adult worms of *Trichostrongylus vitrines* [55]. Analysis of transcriptomic data reveals the presence of these proteases in different developmental stages of *Trichostrongylus colubriformis* maintained *in* culture [56]. These peptidases may play key roles in several physiological processes, such as tissue penetration, immune evasion and feeding.

Our data reveals previously unrecognized peptidase activity which are present in *A*. *costaricensis* at several developmental stages. These results are a first but critical step to our further understanding of the biology and pathogenesis of this nematode.

## Supporting information

S1 File Raw data from the multiplex peptide cleavage assay. These results were used to determine the cleavage site specificities and to build the Icelogo plots related to the peptidase activity found in *A*. *costaricensis* worms.
(XLSX)

S1 Table Panel of fluorogenic substrates used to characterize the endopeptidase activity of *A*. *costaricensis* adult worm lysates at pH 5.0 and 8.0. (XLSX)

S2 Table Fluorogenic substrates used to characterize the aminopeptidase activity of *A*. *costaricensis* adult worm lysates at pH 8.0. (XLSX)

S3 Table Panel of fluorogenic substrates used to determine the aminopeptidase enzyme activity of *A*. *costaricensis* L1 larvae lysates at pH 5.0 and 8.0. (XLSX)

## References

[1] Morera P, Cespedes R. Angiostrongilosis abdominal. Uma nueva parasitosis humana. Acta Med Costarric. 1971;(14):173–89.

[2] Romero-Alegría A, Belhassen-García M, Velasco-Tirado V, Garcia-Mingo A, Alvela-Suárez L, Pardo-Lledias J, et al *Angiostrongylus costaricensis*: systematic review of case reports. Advances in Infectious Diseases 2014;4:36–41. 10.4236/aid.2014.41007

[3] Spratt D. Species of *Angiostrongylus* (Nematoda: Metastrongyloidea) in wildlife: A review Int J Parasitol Parasites Wildl. 2015;4(2):178–89. 10.1016/j.ijppaw.2015.02.006 . 25853051 PMC4381133

[4] Morera P. Life history and redescription of *Angiostrongylus costaricensis* Morera and Cespedes, 1971. Am J Trop Med Hyg. 1973;22(5):613–21. . 4729741 10.4269/ajtmh.1973.22.613

[5] Thiengo SC. Mode of Infection of *Sarasinula marginata* (Mollusca) with Larvae of *Angiostrongylus costaricensis* (Nematoda). Mem Inst Oswaldo Cruz. 1996;91 (3):277–8. 10.1590/S0074-02761996

[6] Mendonça CL, Carvalho OS, Mota EM, Pelajo-Machado M, Caputo LF, Lenzi HL. Penetration sites and migratory routes of *Angiostrongylus costaricensis* in the experimental intermediate host (*Sarasinula marginata*). Mem Inst Oswaldo Cruz. 1999;94:549–56. . 10446018 10.1590/s0074-02761999000400022

[7] Mota EM, Lenzi HL. *Angiostrongylus costaricensis* life cycle: a new proposal. Mem Inst Oswaldo Cruz. 1995;90(6):707–9. . 8731366 10.1590/s0074-02761995000600010

[8] Mota EM, Lenzi HL. *Angiostrongylus costaricensis*: complete redescription of the migratory pathways based on experimental *Sigmodon hispidus* infection. Mem Inst Oswaldo Cruz. 2005;100(4):407–20. S0074-02762005000400012 . 16113890 10.1590/s0074-02762005000400012

[9] Graeff-Teixeira C, Camillo-Coura L, Lenzi HL. Histopathological criteria for the diagnosis of abdominal angiostrongyliasis. Parasitol Res. 1991;77(7):606–11. . 1792232 10.1007/BF00931023

[10] Graeff-Teixeira C, Agostini AA, Camillo-Coura L, Ferreira-da-Cruz MF. Seroepidemiology of abdominal angiostrongyliasis: the standardization of an immunoenzymatic assay and prevalence of antibodies in two localities in southern Brazil. Trop Med Int Health. 1997;2(3):254–60. . 9491104 10.1046/j.1365-3156.1997.d01-266.x

[11] Geiger SM, Laitano AC, C S-T, A A. A, H S-K, Graeff-Teixeira C. Detection of the acute phase of abdominal angiostrongyliasis with a parasite-specific IgG enzyme linked immunosorbent assay. Mem Inst Oswaldo Cruz 2001;96(515–518). . 11391424 10.1590/s0074-02762001000400012

[12] Morera P, Bontempo I. Acción de algunos antihelminticos sobre *Angiostrongylus costaricensis*. Rev Med Hosp Nac Niños (Costa Rica) 1985;20:165–74.

[13] Terada M, Kino H, Akyol CV, Sano M. Effects of mebendazole on *Angiostrongylus costaricensis* in mice, with special reference to the timing of treatment. Parasitol Res. 1993;79(6):441–3. . 8415554 10.1007/BF00931579

[14] Tungtrongchitr A, Ishih A, Terada M, Radomyos P. Effects of sensitization on efficacy of mebendazole in mice infected with adult *Angiostrongylus costaricensis*. Trop Med Parasitol. 1993;44(4):322–6. . 8134774

[15] Mentz MB, Graeff-Teixeira C. Drug trials for treatment of human angiostrongyliasis. Rev Inst Med Trop Sao Paulo. 2003;45(4):179–84. S0036-46652003000400001. . 14502343 10.1590/s0036-46652003000400001

[16] Bohrer Mentz M, Dallegrave E, Agostini A, Graeff-Teixeira C. Phenantroline, lovastatin, and mebendazole do not inhibit oviposition in the murine experimental infection with *Angiostrongylus costaricensis*. Parasitol Res. 2007;100(2):379–82. 10.1007/s00436-006-0271-3 . 16944203

[17] Rodriguez R, Porto SM, Dos Santos Ferrari R, Marcolan AM, da Silva AC, Graeff-Teixeira C, et al Outcomes in mice with abdominal angiostrongyliasis treated with enoxaparin. Parasitol Res 2011;109:787–92. 10.1007/s00436-011-2324-5 . 21400113

[18] Fante CA, Dieterish S, Rodriguez R. Betamethasone and aqueous extract of *Arctium lappa* for treating angiostrongyliasis. Rev Soc Bras Med Trop. 2008;41(6):654–7. S0037-86822008000600018. . 19142447 10.1590/s0037-86822008000600018

[19] Drag M, Salvesen GS. Emerging principles in protease-based drug discovery. Nat Rev Drug Discov. 2010;9(9):690–701. nrd3053 10.1038/nrd3053 . 20811381 PMC2974563

[20] Jilkova A, Horn M, Rezacova P, Maresova L, Fajtova P, Brynda J, et al Activation route of the *Schistosoma mansoni* cathepsin B1 drug target: structural map with a glycosaminoglycan switch. Structure. 2014;22(12):1786–98. S0969-2126(14)00322-0. 10.1016/j.str.2014.09.015 . 25456815

[21] Gonzalez-Bacerio J, Fando R, del Monte-Martinez A, Charli JL, Chavez Mde L. *Plasmodium falciparum* M1-aminopeptidase: a promising target for the development of antimalarials. Curr Drug Targets. 2014;15(12):1144–65. CDT-EPUB-63030. . 25341419 10.2174/1389450115666141024115641

[22] Bibo-Verdugo B, Jiang Z, Caffrey CR, O'Donoghue AJ. Targeting proteasomes in infectious organisms to combat disease. FEBS J. 2017;284(10):1503–17. 10.1111/febs.14029 . 28122162

[23] Nelson FBL, Brown GP, Shilton C, Shine R. Host–parasite interactions during a biological invasion: The fate of lungworms *(Rhabdias spp*.) inside native and novel anuran hosts. Int J Parasitol Parasites Wildl. 2015;4:206–15. 10.1016/j.ijppaw.2015.04.001 . 25973392 PMC4427737

[24] Sansri V, Changklungmoa N, Chaichanasak P, Sobhon P, Meemon K. Molecular cloning, characterization and functional analysis of a novel juvenile-specific cathepsin L of *Fasciola gigantica*. Acta Trop. 2013;128(1):76–84. S0001-706X(13)00168-X. 10.1016/j.actatropica.2013.06.013 . 23820262

[25] Wu XJ, Sabat G, Brown JF, Zhang M, Taft A, Peterson N, et al Proteomic analysis of *Schistosoma mansoni* proteins released during in vitro miracidium-to-sporocyst transformation. Mol Biochem Parasitol. 2009;164(1):32–44. S0166-6851(08)00257-0. 10.1016/j.molbiopara.2008.11.005 . 19095013 PMC2665799

[26] McKerrow JH, Caffrey C, Kelly B, Loke P, Sajid M. Proteases in parasitic diseases. Annu Rev Pathol. 2006;1:497–536. 10.1146/annurev.pathol.1.110304.100151 . 18039124

[27] Rebello KM, Siqueira CR, Ribeiro EL, Valente RH, Mota EM, Perales J, et al Proteolytic activity in the adult and larval stages of the human roundworm parasite *Angiostrongylus costaricensis*. Mem Inst Oswaldo Cruz. 2012;107(6):752–9. S0074-02762012000600008. . 22990964 10.1590/s0074-02762012000600008

[28] O'Donoghue AJ, Eroy-Reveles AA, Knudsen GM, Ingram J, Zhou M, Statnekov JB, et al Global identification of peptidase specificity by multiplex substrate profiling. Nat Methods. 2012;9(11):1095–100. 10.1038/nmeth.2182 . 23023596 PMC3707110

[29] Dvorak J, Fajtova P, Ulrychova L, Leontovyc A, Rojo-Arreola L, Suzuki BM, et al Excretion/secretion products from *Schistosoma mansoni* adults, eggs and schistosomula have unique peptidase specificity profiles. Biochimie. 2016;122:99–109. 10.1016/j.biochi.2015.09.025 . 26409899 PMC4747843

[30] Goupil LS, Ivry SL, Hsieh I, Suzuki BM, Craik CS, O'Donoghue AJ, et al Cysteine and Aspartyl Proteases Contribute to Protein Digestion in the Gut of Freshwater Planaria. PLoS Negl Trop Dis. 2016;10(8):e0004893 10.1371/journal.pntd.0004893 . 27501047 PMC4976874

[31] Colaert N, Helsens K, Martens L, Vandekerckhove J, Gevaert K. Improved visualization of protein consensus sequences by iceLogo. Nat Methods. 2009;6(11):786–7. nmeth1109-786. 10.1038/nmeth1109-786 . 19876014

[32] Ivry SL, Meyer NO, Winter MB, Bohn MF, Knudsen GM, O'Donoghue AJ, et al Global substrate specificity profiling of post-translational modifying enzymes. Protein Sci. 2018;27(3):584–94. 10.1002/pro.3352 . 29168252 PMC5818756

[33] Yasuda Y, Kageyama T, Akamine A, Shibata M, Kominami E, Uchiyama Y, et al Characterization of new fluorogenic substrates for the rapid and sensitive assay of cathepsin E and cathepsin D. J Biochem. 1999;125(6):1137–43. . 10348917 10.1093/oxfordjournals.jbchem.a022396

[34] Lopez-Otin C, Bond JS. Proteases: multifunctional enzymes in life and disease. J Biol Chem. 2008;283(45):30433–7. R800035200. 10.1074/jbc.R800035200 . 18650443 PMC2576539

[35] Sojka D, Hartmann D, Bartosova-Sojkova P, Dvorak J. Parasite Cathepsin D-Like Peptidases and Their Relevance as Therapeutic Targets. Trends Parasitol. 2016;32(9):708–23. 10.1016/j.pt.2016.05.015 . 27344362

[36] Ross B, Krapp S, Augustin M, Kierfersauer R, Arciniega M, Geiss-Friedlander R, et al Structures and mechanism of dipeptidyl peptidases 8 and 9, important players in cellular homeostasis and cancer. Proc Natl Acad Sci U S A. 2018;115(7):E1437–E45. 10.1073/pnas.1717565115 . 29382749 PMC5816189

[37] Robinson MW, Dalton JP, Donnelly S. Helminth pathogen cathepsin proteases: it's a family affair. Trends Biochem Sci. 2008;33(12):601–8. S0968-0004(08)00207-7. 10.1016/j.tibs.2008.09.001 . 18848453

[38] Williamson AL, Lustigman S, Oksov Y, Deumic V, Plieskatt J, Mendez S, et al *Ancylostoma caninum* MTP-1, an astacin-like metalloprotease secreted by infective hookworm larvae, is involved in tissue migration. Infect Immun. 2006;74(2):961–7. 74/2/961. 10.1128/IAI.74.2.961-967.2006 . 16428741 PMC1360348

[39] Yang Y, Wen Y, Cai YN, Vallee I, Boireau P, Liu MY, et al Serine proteases of parasitic helminths. Korean J Parasitol. 2015;53(1):1–11. 10.3347/kjp.2015.53.1.1 . 25748703 PMC4384789

[40] Choe Y, Leonetti F, Greenbaum DC, Lecaille F, Bogyo M, Bromme D, et al Substrate profiling of cysteine proteases using a combinatorial peptide library identifies functionally unique specificities. J Biol Chem. 2006;281(18):12824–32. M513331200. 10.1074/jbc.M513331200 . 16520377

[41] Sajid M, McKerrow JH. Cysteine proteases of parasitic organisms. Mol Biochem Parasitol. 2002;120(1):1–21. S0166685101004388. . 11849701 10.1016/s0166-6851(01)00438-8

[42] Cheng M, Yang X, Li Z, He H, Qu Z, He A, et al Cloning and characterization of a novel cathepsin B-like cysteine proteinase from *Angiostrongylus cantonensis*. Parasitol Res. 2012;110(6):2413–22. 10.1007/s00436-011-2780-y . 22215189

[43] Cezari MH, Puzer L, Juliano MA, Carmona AK, Juliano L. Cathepsin B carboxydipeptidase specificity analysis using internally quenched fluorescent peptides. Biochem J. 2002;368(Pt 1):365–9. 10.1042/BJ20020840 . 12201820 PMC1222986

[44] Yu C, Wang Y, Zhang J, Fang W, Luo D. Immunolocalization and developmental expression patterns of two cathepsin B proteases (AC-cathB-1, -2) of *Angiostrongylus cantonensis*. Exp Parasitol. 2014;144:27–33. Epub 2014/06/15. 10.1016/j.exppara.2014.06.008 . 24929149

[45] Caffrey CR, Goupil L, Rebello KM, Dalton JP, Smith D. Cysteine proteases as digestive enzymes in parasitic helminths. PLoS Negl Trop Dis. 2018;12(8):e0005840 10.1371/journal.pntd.0005840 . 30138310 PMC6107103

[46] Park JN, Park SK, Cho MK, Park MK, Kang SA, Kim DH, et al Molecular characterization of 45 kDa aspartic protease of *Trichinella spiralis*. Vet Parasitol. 2012;190(3–4):510–8. S0304-4017(12)00343-3 10.1016/j.vetpar.2012.06.029 . 22795939

[47] Jolodar A, Fischer P, Buttner DW, Miller DJ, Schmetz C, Brattig NW. *Onchocerca volvulus*: expression and immunolocalization of a nematode cathepsin D-like lysosomal aspartic protease. Exp Parasitol. 2004;107(3–4):145–56. 10.1016/j.exppara.2004.06.006 . 15363940

[48] Sojka D, Franta Z, Horn M, Hajdusek O, Caffrey CR, Mares M, et al Profiling of proteolytic enzymes in the gut of the tick *Ixodes ricinus* reveals an evolutionarily conserved network of aspartic and cysteine peptidases. Parasit Vectors. 2008;1(1):7 10.1186/1756-3305-1-7 . 18348719 PMC2289814

[49] Williamson AL, Brindley PJ, Abbenante G, Prociv P, Berry C, Girdwood K, et al Cleavage of hemoglobin by hookworm cathepsin D aspartic proteases and its potential contribution to host specificity. FASEB J. 2002;16(11):1458–60. 10.1096/fj.02-0181fje . 12205047

[50] Williamson AL, Brindley PJ, Loukas A. Hookworm cathepsin D aspartic proteases: contributing roles in the host-specific degradation of serum proteins and skin macromolecules. Parasitology. 2003;126(Pt 2):179–85. . 12636356 10.1017/s0031182002002706

[51] Williamson AL, Lecchi P, Turk BE, Choe Y, Hotez PJ, McKerrow JH, et al A multi-enzyme cascade of hemoglobin proteolysis in the intestine of blood-feeding hookworms. J Biol Chem. 2004;279(34):35950–7. 10.1074/jbc.M405842200 . 15199048

[52] Delcroix M, Sajid M, Caffrey CR, Lim KC, Dvorak J, Hsieh I, et al A multienzyme network functions in intestinal protein digestion by a platyhelminth parasite. J Biol Chem. 2006;281(51):39316–29. Epub 2006/10/10. M607128200 10.1074/jbc.M607128200 . 17028179

[53] Sakanari JA, McKerrow JH. Identification of the secreted neutral proteases from *Anisakis simplex*. J Parasitol. 1990;76(5):625–30. . 2213405

[54] Hong X, Bouvier J, Wong MM, Yamagata GY, McKerrow JH. *Brugia pahangi*: identification and characterization of an aminopeptidase associated with larval molting. Exp Parasitol. 1993;76(2):127–33. . 8454021 10.1006/expr.1993.1015

[55] MacLennan K, Gallagher MP, Knox DP. Stage-specific serine and metallo-proteinase release by adult and larval *Trichostrongylus vitrinus*. Int J Parasitol. 1997;27(9):1031–6. S002075199700074X. . 9363485 10.1016/s0020-7519(97)00074-x

[56] Cantacessi C, Mitreva M, Campbell BE, Hall RS, Young ND, Jex AR, et al First transcriptomic analysis of the economically important parasitic nematode, *Trichostrongylus colubriformis*, using a next-generation sequencing approach. Infect Genet Evol. 2010;10(8):1199–207. S1567-1348(10)00212-1. 10.1016/j.meegid.2010.07.024 . 20692378 PMC3666958

